# The role of dietary sugars, overweight, and obesity in type 2 diabetes mellitus: a narrative review

**DOI:** 10.1038/s41430-022-01114-5

**Published:** 2022-03-21

**Authors:** Meike Veit, Renske van Asten, Andries Olie, Philip Prinz

**Affiliations:** 1 Department Consumer Protection—Nutrition Policy—Sustainability Europe, Pfeifer & Langen GmbH & Co. KG, Cologne, Germany; 2 Knowledge Centre Sugar & Nutrition, Hilversum, The Netherlands; 3 Board Members, Knowledge Centre Sugar & Nutrition, Hilversum, The Netherlands; 4 Royal Cosun, Breda, The Netherlands; 5 Department of Nutritional Sciences, German Sugar Association, Berlin, Germany

**Keywords:** Type 2 diabetes, Obesity

## Abstract

Nowadays, there is still a popular belief that dietary sugars, in particular sucrose, are directly linked to the development of type 2 diabetes mellitus (T2DM). Furthermore, since insulin action is impaired in T2DM, it is still believed that excluding dietary sugars from the diet can adequately treat T2DM. This might be based on the assumption that dietary sugars have a stronger impact on blood glucose levels than other carbohydrates. Therefore, the aim of this review is to discuss the effects of dietary sugars intake, including sugar-sweetened beverages (SSBs) against the background of overall energy intake and weight gain in the development of T2DM. Furthermore, the effect of dietary sugars, including SSBs on glycemic control will be discussed. Results from various systematic reviews and meta-analyses do not support the idea that the intake of sucrose and other dietary sugars is linked to T2DM. Long-chain or complex carbohydrates can have a greater impact on postprandial glycemic response than sucrose. SSBs do not affect glycemic control if substituted for other calorie sources. Current scientific evidence clearly points toward excess energy intake followed by excess body fat gain being most relevant in the development of T2DM.

## Introduction

Globally, the prevalence of overweight and obesity has risen in the last decades [1], obesity, in particular, has doubled in more than 70 countries since 1980 and at the same time so has the risk of several non-communicable diseases (NCDs), including type 2 diabetes mellitus (T2DM) [2]. The worldwide diabetes prevalence in adults has increased from 4.3% to 9.0% in men and from 5.0% to 7.9% in women [3]. Although this rise includes both type 1 diabetes mellitus (T1DM) and T2DM, it is expected that nearly 85% to 95% of this increase is due to the incidence of T2DM [3].

Although it is well-known that T2DM is a multifactorial disease with diverse risk factors, including lifestyle factors such as smoking, a lack of exercise, and a poor diet [4], the role of dietary sugars, especially sucrose, is still controversially discussed. This might be due to the assumption that sucrose and other dietary sugars induce a stronger postprandial glycemic response than longer-chained carbohydrates [5, 6].

However, overweight and obesity are major risk factors for T2DM since body weight gain increases the risk of T2DM, especially when accompanied by excess body fat gain [7, 8]. Obesity leads to increased lipid storage in adipose tissues, which at a certain point no longer have the capacity to store excess energy intake. At this point, adipose tissues release free fatty acids (FFAs) by increased lipolysis, which remain in circulation [9] and result in allover increased circulating levels of FFAs [8, 10], which in turn promote muscle and hepatic insulin resistance (IR) as well as impaired insulin secretion of the ß-cell in the pancreas [7, 8]. Although the exact mechanism by which lipids induce IR is still under debate, the most prominent hypothesis is that intracellular lipid metabolites cause defects in insulin signaling and consequently attenuate glucose uptake in skeletal muscle and adipose cells as well as increase hepatic glucose production and decrease hepatic glycogen synthesis, which results in hyperglycemia [8, 11]. A brief description and corresponding figure of insulin action under normal and obese conditions can be found in the supplementary information (Supplementary Fig. 1).

Nevertheless, dietary sugars are still debated to cause T2DM independently from energy intake [12]. Therefore, the aim of this review is to discuss the effects of dietary sugar intake in the development of T2DM as well as on glycemic control against the background of excess energy intake and obesity. The results for the current review were derived from systematic reviews and meta-analyses of observational as well as intervention studies, which provide the highest scientific evidence. If data was not available from systematic reviews and meta-analyses, data from intervention studies, mainly randomized controlled trials (RCTs), was used to further ensure the high scientific quality. This review only contains results from human studies and did not use results from animal studies or cell experiments.

## Definitions of dietary sugars

One reason for the assumption that dietary sugars consumption is directly linked to the development of T2DM might be that the definitions of different types of sugars are not used properly.

There are three main definitions of dietary sugars: (1) “added sugars”, including all mono- and disaccharides that are added to foods during processing and preparation; (2) “free sugars” which, according to the definition of the World Health Organization (WHO), comprise all mono- and disaccharides that are added to foods by the manufacturer, cook or the consumer as well as the sugars that are naturally present in honey, syrups, and fruit juices; and (3) “total sugars”, which include all mono- and disaccharides that naturally occur in food as well as added mono- and disaccharides [13]. These three definitions naturally include all monosaccharides (e.g., glucose, fructose, and galactose) as well as all disaccharides (e.g., sucrose, lactose, and maltose) [13].

To avoid further confusion of sugars definitions, this review will refer exactly to the definitions used in the original works.

## Dietary sugars, glycemic control, and T2DM

For sucrose, there is currently no scientific data, which allows the conclusion to be drawn that it has a detrimental effect on glycemic control and T2DM. Isocaloric exchange of sucrose with other carbohydrates did not affect glycemic control [14–17], including HbA1c-level (a marker of glycated hemoglobin, reflecting long-term average blood glucose levels) [15, 16] in patients with T2DM even with large intakes of sucrose (220 g/d) [16]. Additionally, a recently published network meta-analysis of RCTs showed no effect on HbA1c level following isocaloric replacements of glucose, fructose, or sucrose with starch [18], clearly indicating no detrimental effect on glycemic control compared to other carbohydrates. These results are further supported by systematic reviews and meta-analyses of prospective cohort studies, which showed no association of total sugars and fructose but a small inverse association of sucrose intake (50–78 g/d) with T2DM incidence [19, 20]. These results show that sucrose intake is associated with a decreased risk of T2DM.

Taken together, current scientific evidence from observational and intervention studies does not allow the conclusion to be drawn that sucrose, glucose, or fructose are linked to T2DM or have a detrimental effect on glycemic control. However, it has to be kept in mind that a hypercaloric diet, which can be achieved by overconsumption of any type of macronutrients, increases body weight, which in turn is an established risk factor for the development of T2DM. Indeed, a hypercaloric dietary sugars intake can increase body weight as well, an effect that may be more attributable to excess energy intake than dietary sugars per se [21].

## SSBs, glycemic control, and T2DM

Although current scientific evidence indicates no association between dietary sugars and the development of T2DM, the role of sugar-sweetened beverages (SSBs) in the development of T2DM is the subject of controversial discussion. Several systematic reviews and meta-analyses of prospective cohort studies indicate that a higher SSB consumption (≥1 serving per day) is associated with an increased risk of T2DM [20]. Because prospective cohort studies cannot show causality, it is possible that high SSB consumption contributes to the development of T2DM by providing extra calories to the normal diet. This is further supported by a recently published systematic review and meta-analysis of RCTs by Choo et al. [22], showing that SSBs do not have an adverse effect on glycemic control if isocalorically exchanged for other calorie sources (including HbA1c, fasting blood glucose, and fasting insulin level). SSBs only have an adverse effect on glycemic control when adding excess energy to the diet. At this stage, it has to be mentioned that, in the pyramid of evidence-based medicine, the results of observational prospective studies are rated lower than the results of RCTs, because only RCTs can show causality [23]. The main problem of observational prospective cohort studies is the difficulty in isolating the effect of one dietary factor from all the other dietary factors as well as lifestyle factors regarding the development of T2DM. T2DM is also linked to several dietary and lifestyle factors, including smoking, physical activity, alcohol, and coffee consumption as well as consumption of red meat [24, 25].

Additionally, people who consume high amounts of SSBs are more likely to have a higher energy intake, be less physically active, and smoke more [19]. It is impossible to exclude all these confounders in prospective cohort studies to isolate the direct effect of one single dietary factor, in this case, the direct association of SSBs on T2DM [19]. Therefore, the findings by Choo and colleagues with the highest scientific evidence from a systematic review and meta-analysis of RCTs clearly indicate that excess energy intake rather than SSB intake per se increases the risk for the development of T2DM.

Furthermore, it should be mentioned that SSBs have a special physiological role regarding hunger and satiety. In general, liquid calories (regardless of the predominant macronutrient source) have a less pronounced effect on satiety compared to solid calories, resulting in a faster recurrence of hunger and hence an increase in the risk of overall increased energy intake [26, 27]. All in all, SSBs can contribute to an overall higher energy intake due to their less pronounced effect on satiety. Since SSBs do not affect body weight [21] or glycemic control [22] if isocalorically exchanged for other macronutrients, it may be assumed that the association of SSBs and T2DM by findings from prospective cohort studies is more a result of excess energy intake than of SSBs per se.

## Dietary sugars and postprandial glycemic response

Dietary sugars, such as sucrose, because of their short chain of two sugar molecules, are believed to induce a stronger postprandial glycemic response and hence greater insulin secretion than other carbohydrates with more complex chains. Contrary to this assumption, various analyses of different carbohydrates showed that postprandial glycemic responses differ widely and are dependent on various factors, including the amount of fiber or fat content in foods, but especially the grade of processing of carbohydrates in foods, which results in easier enzymatic accessibility, faster digestion and consequently to a sharper increase in blood glucose level [28, 29]. Several studies showed that diverse carbohydrates induce stronger postprandial glycemic responses than dietary sugars, disproving the hypothesis that dietary sugars, such as sucrose, lead to one of the strongest postprandial glycemic responses [28, 30]. Additionally, there is also the assumption that complex carbohydrates lead to a less pronounced rise in postprandial glycemic responses and therefore should be preferred. In contrast to this, the branched-chain carbohydrate “amylopectin” leads to a more rapid increase in blood glucose, whereas “amylose” (an unbranched carbohydrate) has a less pronounced effect on blood glucose levels [31]. These findings indicate that the complexity and length of carbohydrates are not proper indicators to describe the postprandial glycemic response and thus show the need for a more individual approach to the rating of carbohydrates or carbohydrate-containing foods regarding their effect on the blood glucose level. Furthermore, the assumption that sucrose (and also other dietary sugars) induces a strong postprandial glycemic response, because of its short chain of two molecules is incorrect and cannot be confirmed in scientific analysis.

## Glycemic index (GI)

The glycemic index (GI) is a well-known measure for assessing the postprandial glycemic response due to its strong correlation with postprandial glucose concentration [28, 32]. With reference to Atkinson and colleagues, a high GI is defined as being 70 or greater and can be related to various bread, breakfast cereals, or rice, whereas a low GI is 55 or less and can be related to various legumes, pasta, fruits, or dairy products [33]. Sucrose has a medium GI of 65 [33] because it is half glucose and half fructose. Fructose has a different metabolic pathway compared to glucose and needs to be converted into glucose (approximately 50% of the fructose) within hepatocytes first, before the newly formed glucose is released into the blood circulation [34]. Therefore, fructose shows only minimal changes in blood glucose level and has a very low GI of 15, whereas carbohydrates with longer or more complex chains that consist only of glucose can have a greater impact on postprandial glycemic response [28, 33]. This is important when it comes to food reformulation and sucrose is replaced by other carbohydrates. In this case, the calorie content of the reformulated food is still the same, but the effect on glycemic control might be stronger than before.

## Glycemic load (GL)

GI is a standardized value for blood glucose response and is an indicator for the carbohydrate-containing food itself, representing its quality. However, GI does not take into consideration the content of carbohydrates in the amount of food that is consumed. Therefore, glycemic load (GL) was proposed as a measure to quantify and compare the effects of carbohydrate-containing foods or diets. GL is the product of GI and the carbohydrate content of a certain food divided by the serving size, usually in grams (GL = GI × carbohydrate content/serving size in g) [35]. A high GL is defined as 20 or greater and a low GL as 10 or less. In between (less than 20, but more than 10) GL is medium [36]. The association of GI and GL is not necessarily straightforward, there are foods with a low GI but a high GL, e.g., different types of noodles or pasta, usually eaten in greater amounts, and foods with a low GL but a high GI, e.g., a watermelon, if eaten in a small portion size [33, 36].

## GI, GL, and T2DM

The effectiveness of using GI/GL as dietary parameters in the treatment and development of T2DM is still under dispute. Two systematic reviews and meta-analyses of prospective cohort studies showed that a high GI/GL is associated with an increased incidence of T2DM [20, 37]. Very recently, Livesey and colleagues performed a systematic review and meta-analysis of prospective cohort studies, indicating that persons consuming a diet with an average GI of 76 could have an 87% higher risk of developing T2DM than people consuming a diet with a dietary GI of 48. Additionally, people consuming a diet with a GL of 257 g/d in a 2000 kcal intake (which is the sum of GLs per food eaten per day for people consuming 2000 kcal) could have an 89% higher risk of T2DM than people consuming a diet with a GL of 73 g/d in 2000 kcal [38]. However, a systematic review of current literature pointed out that current data from prospective cohort studies is controversial. Although there are several studies, which showed an increased T2DM risk with increasing GI/GL, there are many prospective cohort studies, which showed no association between GI/GL and the risk of T2DM with a follow-up of 12 or more years [39]. Since prospective cohort studies can only show correlation but not causality, findings of intervention studies are needed to underline the relevance of GI/GL in the treatment and development of T2DM.

A very recent systematic review and meta-analysis of RCTs showed that a diet with a low GI/GL compared to higher GI/GL control diets (Median low vs. high: GI 49 vs 63 and GL 102 vs. 138) in overweight and obese patients with T1DM and T2DM reduced HbA1c-level, fasting glucose, but not blood insulin [40], indicating that a diet with a low GI/GL can be useful for overweight and obese patients with T2DM. For diets with a low GI, these findings were further confirmed in a systematic review of RCTs, indicating that lowering the GI in the diet seems to be an effective method of improving glycemic control in diabetes [41]. In the systematic review and meta-analyses of Chiavaroli and colleagues, body weight decreased in the low GI/GL groups [40]. However, data were not adjusted for total energy intake, consequently, reduction in energy intake due to the intervention in the participants’ diet could not be excluded.

Diet and lifestyle remain the cornerstone of the management of diabetes, which is also confirmed by a very recent umbrella review of published meta-analyses of RCTs, which showed that hypocaloric diets for weight management in people with T2DM are superior for remission compared to diets that focus on any particular macronutrient profile or style, including low GI diets [42]. Taken together, since overweight and obesity are major risk factors for T2DM [7, 8], a reduction in body weight, more specifically body fat loss, due to reduction of total energy intake should be the primary goal in the treatment of T2DM but a diet with a low GI/GL could also be beneficial for overweight and obese patients with T2DM.

## Body weight and T2DM

It is well-known that increasing overweight and obesity is the most important risk factor for T2DM [7, 8] and that weight loss can reverse T2DM pathophysiology and improve glycemic control [43].

The importance of the degree of body weight reduction in T2DM was reflected by the DiRECT (Diabetes Remission Clinical Trial) study, which showed that weight reduction in subjects having overweight and obesity, as well as T2DM, can lead to remission (defined as a level of glycemia below the diagnostic threshold of HbA1c < 48 mmol/mol or 6.5% in the absence of pharmacological or surgical interventions [44]) of T2DM and was proportional to body weight loss in a period of 12 months [45]. The greater the weight loss increases the greater the chances for T2DM remission with a remission rate of 86% in patients that lost 15 kg or more [45].

In line with these findings, lifestyle changes focusing on individuals’ diet and physical activity are most promising in T2DM treatment. A recently published systematic review and meta-analysis of RCTs showed that body weight reduction due to increased physical activity and dietary changes (mainly energy intake reduction) is the cornerstone in the prevention of T2DM [46]. These findings are further supported by independent systematic reviews and meta-analyses of intervention studies, demonstrating that body weight reduction due to restriction of energy intake and lifestyle changes improves glycemic control in patients with T2DM [47, 48]. Body weight reduction due to hypocaloric diets normalized hyperglycemia by improving ß-cell function as well as hepatic IR in subjects having obesity and T2DM [49, 50] and also improved IR in skeletal muscle in young, lean, insulin-resistant subjects by promoting insulin-stimulated muscle glucose uptake [51].

Taken together, there is consistent scientific evidence showing that body weight reduction due to reduced energy intake and lifestyle changes, including increased physical activity are key to improving glycemic control and hence promoting T2DM remission. A hypocaloric diet to reduce body weight can be achieved by the reduction of any type of macronutrients. Indeed, hypocaloric dietary sugars intake can reduce body weight as well, an effect that is more attributable to reduced energy intake than dietary sugars per se [21].

## Conclusions

The major risk factor for T2DM, although it is a multifactorial disease, is a positive energy balance, mainly due to increased energy intake and reduced physical activity, resulting in overweight and obesity. It is not sucrose or other dietary sugars per se but elevated circulating FFAs due to excessive body weight that induces IR in skeletal muscle and the liver, resulting in hyperglycemia and providing the first steps in the development of T2DM. Although current data from observational studies provide evidence that SSBs are linked to T2DM, controlled intervention studies with the highest level of scientific evidence did not show any effects of SSBs on glycemic control under isocaloric conditions. Therefore, the effect of SSBs on T2DM seems to be mediated by excess energy intake.

However, there are still open research questions in the field of dietary sugars and T2DM. For example, current data from observational studies clearly show that sucrose and other dietary sugars are not associated with the risk of T2DM, whereas SSBs are. Moreover, sucrose intake shows small inverse associations, which indicates that future research should rather focus on food groups than on single nutrients. Regarding SSBs, long-term interventions studies on their effect on satiety and total energy intake are still missing. These studies are highly needed for a better understanding of the role of SSBs in the development of overweight and obesity as well as T2DM.

## Supplementary information

Supplementary Information

## Data Availability

Data sharing is not applicable to this article as no datasets were generated or analyzed during the current study.
