# Supplementary material for: The role of dietary sugars, overweight, and obesity in type 2 diabetes mellitus: a narrative review
Source: Eur J Clin Nutr. 2022 Mar 21;76(11):1497–501. doi: 10.1038/s41430-022-01114-5 (PMC9630103; doi:10.1038/s41430-022-01114-5)
Supplement: Supplementary file 1 — Supplementary Information [file 41430_2022_1114_MOESM1_ESM.docx]

**Supplementary information Veit et al. 2022**


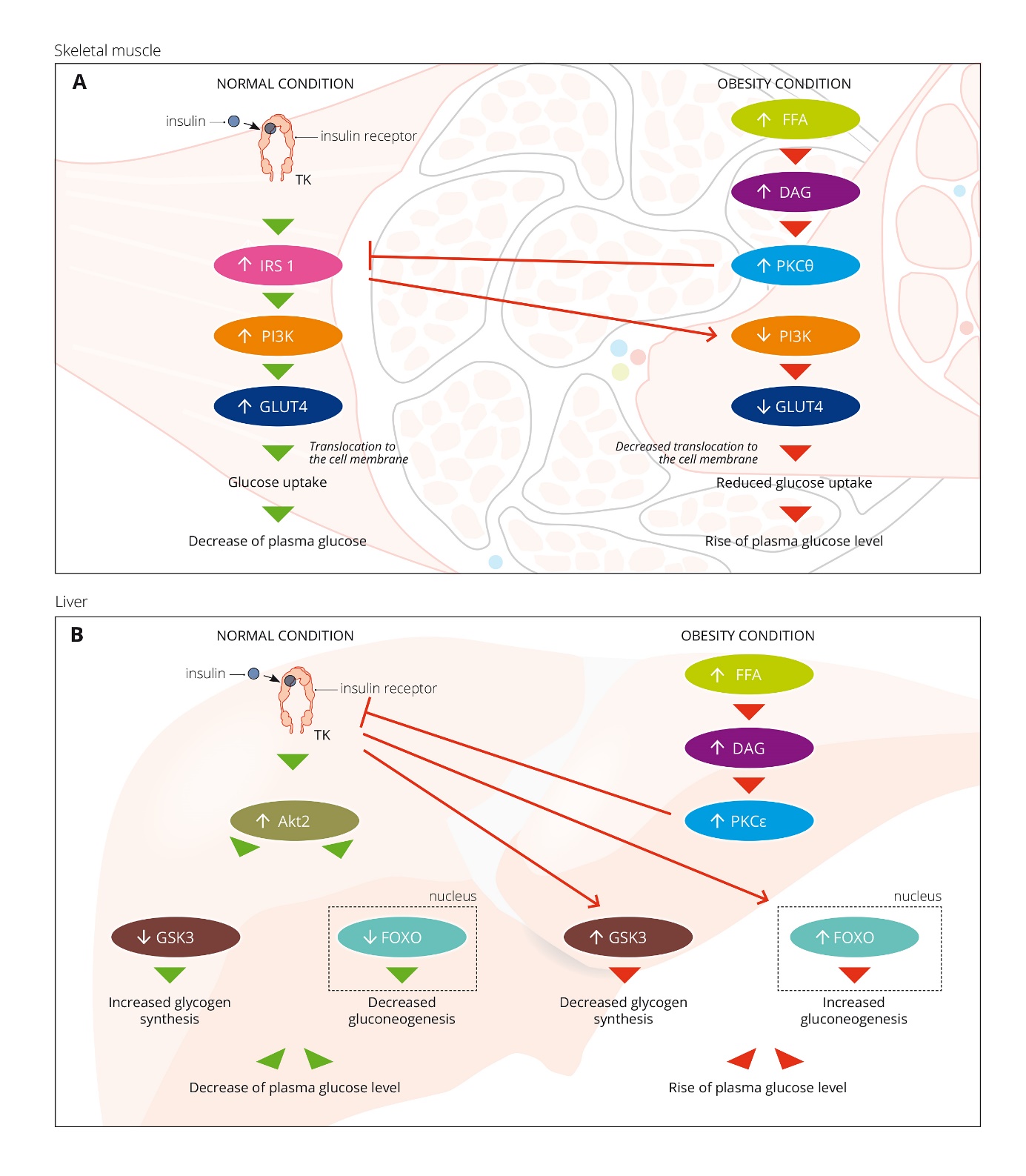


**Supplementary Figure 1:** Under normal conditions, insulin binds to its receptor in skeletal muscle, activating the insulin receptor tyrosine kinase, which results in subsequent phosphorylation of the insulin receptor substrate 1 (IRS1). IRS1 in turn phosphorylates the phosphatidylinositol 3-kinase (PI3K) that promotes the translocation of the glucose transporter type 4 (GLUT4) to the cell membrane and enables glucose uptake in the cell [1]. In obesity, the high level of circulating FFAs leads to a rise in intracellular content of diacylglycerol (DAG) in skeletal muscle. Here, an increased DAG content promotes the activity of protein kinase C θ (PKCθ), which results in increased IRS-1 serine phosphorylation and inhibits the insulin-stimulated tyrosine phosphorylation of IRS-1. This blockade prevents the phosphorylation of Pi3K and consequently decreases GLUT 4 translocation, resulting in reduced glucose uptake [1] [Figure 1A].

In the liver, insulin binds to its receptor and increases glycogen production and decreases gluconeogenesis under normal conditions by activating Akt2 signaling [2]. However, if intracellular DAG content increases, the epsilon isoform of protein kinase C (PKCε) will be activated, which inhibits the insulin receptor tyrosine kinase and hence attenuates hepatic insulin-stimulated glycogen synthesis by activation of glycogen synthase kinase 3 (GSK3). Additionally, this process also decreases phosphorylation of forkhead box subgroup O (FOXO), which translocates into the nucleus, promoting gene expression of gluconeogenic enzymes and hence increasing gluconeogenesis [1] [Figure 1B].

Finally, IR in skeletal muscle and liver followed by impaired insulin secretion of pancreatic ß-cells will result in hyperglycemia, the main characteristic of diabetes [3]. Hyperglycemia promotes several pathophysiological pathways, including the formation of advanced glycation end products and increased formation of intracellular reactive oxygen species, which can contribute to the development of microvascular complications, such as retinopathy, nephropathy or neuropathy as well as macrovascular complications, such as cardiovascular diseases (CVDs), including hypertension or stroke [3,4].

**References**

1. Shulman GI. Ectopic fat in insulin resistance, dyslipidemia, and cardiometabolic disease. N Engl J Med. 2014 Sep 18;371(12):1131–41.

2. Samuel VT, Shulman GI. The pathogenesis of insulin resistance: integrating signaling pathways and substrate flux. J Clin Invest. 2016 Jan;126(1):12–22.

3. DeFronzo RA, Ferrannini E, Groop L, Henry RR, Herman WH, Holst JJ, et al. Type 2 diabetes mellitus. Nat Rev Dis Primer. 2015 23;1:15019.

4. Skyler JS, Bakris GL, Bonifacio E, Darsow T, Eckel RH, Groop L, et al. Differentiation of Diabetes by Pathophysiology, Natural History, and Prognosis. Diabetes. 2017 Feb 1;66(2):241–55.
